# *Hippotherium* Datum implies Miocene palaeoecological pattern

**DOI:** 10.1038/s41598-022-07639-w

**Published:** 2022-03-04

**Authors:** Boyang Sun, Yan Liu, Shanqin Chen, Tao Deng

**Affiliations:** 1 Key Laboratory of Vertebrate Evolution and Human Origins of Chinese Academy of Sciences, Institute of Vertebrate Paleontology and Paleoanthropology, Chinese Academy of Sciences, Beijing, 100044 China; 2 CAS Center for Excellence in Life and Paleoenvironment, Beijing, 100044 China; 3 Hezheng Paleozoological Museum, Hezheng, 731200 Gansu China; 4 University of Chinese Academy of Sciences, Beijing, 100049 China

**Keywords:** Palaeontology, Palaeoecology

## Abstract

Here, we report well–preserved skulls and postcranial specimens of genus *Hippotherium* from the Linxia Basin, Gansu, China. Based on morphological comparison, the species of *Hippotherium* in China, *Hippotherium weihoense* and *Hippotherium chiai*, should be ascribed to the same species, *H*. *weihoense*. We also reviewe other Old World hipparion species in the very early Late Miocene and figure out two evolutionary routes: the *Hippotherium* and *Cormohipparion* lineages. Analysis of locomotive ability indicates that *H*. *weihoense* likely lived in an open habitat, whereas other species of *Hippotherium* likely lived in closed habitats. This result shows a palaeoecological pattern in the early Late Miocene in Eurasia influenced by a series of geological events as aridification of mid–latitude Asia progressed, whereas Europe and North Africa remained relatively humid. As the genus originated from East Asia, hipparion horses divided rapidly into different groups with differing functional morphology to occupy diverse niches.

## Introduction

The dispersal of hipparion horses into the Old World, previously recognised as the *Hipparion* Datum^1–3^, later revised as *Hippotherium* Datum^4,5^, is one of the most significant palaeobiological events in the Late Miocene. *Hippotherium primigenium* in Europe is traditionally regarded as the earliest and most primitive hipparion species in Eurasia^5–7^. Qiu et al.^8^ indicated that all hipparion species in the Old World should be assigned to one genus, *Hipparion*, and that the taxon *Hippotherium* should be regarded as a subgenus. They reviewed the earliest *Hipparion* species found in China, *Hipparion weihoense* and *Hipparion chiai*, and ascribed them to the subgenus *Hippotherium*. They also ascribed other early species to this subgenus. However other authors proposed that *Hippotherium* should be assigned to a valid genus^7,9,10^.

Recent authors proposed the term *Cormohipparion* Datum to reflect that species of *Cormohipparion* represented the first occurrence of hipparion horses in Old World^11,12^. It is necessary to figure out which of *Cormohipparion* Datum or *Hippotherium* Datum is true. Research on classification of *Hippotherium* in China remains insufficient. Liu et al.^13^ erected these two species based on cranial and dental material found in Lantian, Shaanxi. Liu^10^ described another collection from Lantian. However, known specimens were limited to broken skulls, mandibles, isolated teeth, and metapodials. These two species are almost always found in the same locality. They have many morphological similarities, and *H*. *weihoense* specimens are much more abundant than *H*. *chiai*. The actual status of *H*. *chiai* is difficult to determine. Consequently, more and better–preserved specimens are required to evaluate the status of these two species. The evolution and distribution of the earliest hipparion species have great significance regarding the evolution of hipparion horses and Late Miocene palaeoecology. However all of above debates have made it difficult to further investigate this topic.

Recently, we were able to study an excellent collection of specimens of *Hippotherium* from the Linxia Basin, Gansu, China (SI Fig. S1), including a well–preserved skull found at the Shuanggongbei locality (SI Fig. S2); a broken skull accompanied by fore– and hindlimbs from the Niugou locality (SI Figs. S3–S5), and a number of specimens from other localities. These new findings provide more complete information on cranial and postcranial morphology to compare with known specimens of *Hippotherium,* to better characterise *H*. *weihoense* and *H*. *chiai*. This material is also suitable to determine the locomotive ability of *Hippotherium* in China. Deng et al.^14^ performed comprehensive locomotor analysis of the Tibetan *Plesiohipparion zandaense* (their *Hipparion zandaense*), which provides an ideal template for our research. Based on comparison with the known postcranial material of *H*. *primigenium* and *Cormohipparion africanum*, we can seek clues regarding the environmental and ecological conditions of the early Late Miocene in Eurasia.

## Results

### Geological setting

**Figure 1:**
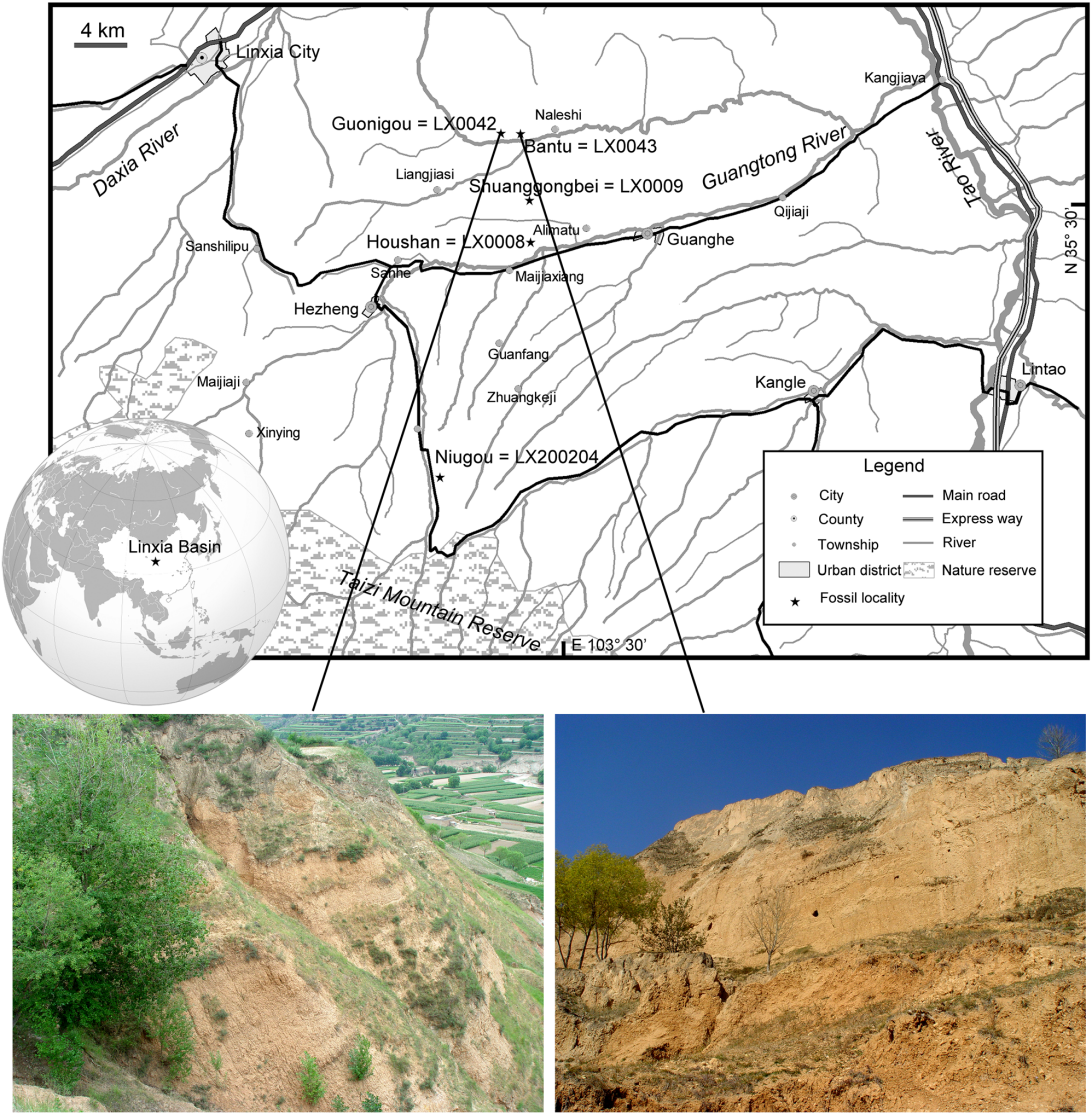
Map and geology of the occurrence area of *H*. *weihoense* in the Linxia Basin, with photos of the most significant localities of Eurasian hipparion (generated with Adobe Photoshop version CS 6, based on the locator map provided by S. Wang, and photographs provided by T. Deng).

The new specimens described were collected from three localities in the Linxia Basin, Gansu, China: Houshan (LX 0008); Shuanggongbei (LX 0009); Niugou (LX 200204) (Fig. 1). Four classic Late Miocene faunas have been erected in the Linxia Basin: Guonigou Fauna (11.5–9.8 Ma), Dashengou Fauna (9.8–8.7 Ma), Yangjiashan Fauna (8.7–7.25 Ma) and Qingbushan Fauna (7.25–5.3 Ma) (after magnetostratigraphic data of Fang et al.^15,16^; and biochronologic comparison by Deng et al.^17^). Based on faunal composition, the species found in Houshan, Shuanggongbei and Niugou relate to the Dashengou Fauna (abundant *Hezhengia bohlini*, *Dinocrocuta giganteum* and *H. weihoense* present, Deng et al.^17^ and this study).

Recently, a new skull has been retrieved in the Bantu locality (Linxia Basin). The Bantu locality is located in the same strata as the Guonigou locality and is biostratigraphically correlative to the Guonigou Fauna^17^. This skull is covered with surrounding rock and needs to be prepared. But some exposed parts show very typical features of *H. weihoense*, such as developed subtriangular preorbital fossa (POF) (SI Fig. S6, i). The recent high-accuracy data of magnetostratigraphy performed by Fang et al.^16^ provided an updated chronological framework for the Linxia Basin, with an absolute age for the lower boundary of the Guonigou Fauna of 11.5 Ma, which is the earliest record for a *Hipparion*-fauna in Eurasia. A potential record was likely present at the Guonigou locality, represented by an isolated M3 with features resembling *H. weihoense*, such as complicated fossette ornamentation and elongated oval protocone, but more proofs are required for certainty.

### Systematic palaeontology

Order Perissodactyla Owen, 1848

Family Equidae Gray, 1821

Genus *Hippotherium* Kaup, 1833

*Hippotherium weihoense* (Liu et al., 1978).

Synonyms

1978: *Hipparion chiai* Liu et al.; Plate 28:1–3, pp. 172–175 (original description)

2005: *Hippotherium primigenium* Zouhri and Bensalmia; Fig. 1D, pp. 63–64.

**Holotype.** A nearly complete skull, the posterior part of the orbit lost, IVPP V 3113.1.

**Type locality.** Shuijiazui, Lantian, Shaanxi (63702.L4).

**Referred specimens**. See SI.

**Revised diagnosis.** Large hipparion horse. Moderately robust muzzle. Shallow nasal notch. Preorbital bar (POB) very long. Preorbital fossa (POF) developed, subtriangular–shaped with distinct anterior margin. Proportion of basal cranial relatively low. Short anterostyle/anterostylid. Developed and complex plications in pre– and postfossettes at middle wear stage. Pli caballin double. Protocone usually elongated at middle wear stage. Deep hypoconal groove at middle wear stage. Protostylid present. Rounded metaconid, subtriangular metastylid with a pointed labial horn. Linguaflexid U–shaped. Pli caballinid present on premolar at middle wear stage. Slender limbs, with metapodials and phalanges III relatively elongated.

**Distribution.** Lantian and Fugu, Shaanxi; Qaidam, Qinghai; and Linxia Basin, Gansu, China.

**Age.** Late Miocene, Bahean, 11.5–7.25 Ma.

### Attribution and revision

The newly described specimens have a characteristic, diagnostic combination of cranial and dentition morphology, including medium to large size (basal length range 410–440 mm; cheek tooth row length range 130–160 mm, Table S1), shallow nasal notch, long POB (length range 40–50 mm, Table S1), developed POF with a posterior pocket, complex fossettes with long and strong folds, and elongated protocone with flat labial margin. All of these features are identical to *Hippotherium weihoense*.

Liu et al.^13^ reported a large hipparion species discovered from Lantian, Shaanxi and erected the new species *Hipparion weihoense*. In the same text, they identified a smaller skull fragment and some teeth with similar features and stratigraphic position to *H*. *weihoense* as another new species *Hipparion chiai*. Qiu et al.^8^ reviewed hipparion fossils from China and accepted the validity of both species. They ascribed these two species to subgenus *Hippotherium*, gave an estimated age of 11–10 Ma to them, and regarded them as those among the most primitive hipparion horses in the Old World. Liu^10^ described a series of specimens from Lantian, Shaanxi and identified some of these specimens as *H*. *weihoense* and others as *H*. *chiai*. Based on the reported specimens from China, there is actually no clear boundary between the cranial features of these two species. The only cranial specimen of *H. chiai* in Lantian is the very fragmentary type skull. Liu et al.^13^ argued that *H. chiai* had an elongated POF. However the facial part of the type skull has been obviously deformed by diagenetic crushing (SI Fig. S6b). In more recent report, Lantian specimens and Fugu ones respectively attributed by Liu^10^ and Li et al.^18^ into *H. chiai* also have subtriangular POFs.

The dentition was regarded as another important feature to distinguish these two species in previous research. Liu et al.^13^ argued that *H*. *chiai* has a simpler fossette ornamentation on the upper cheek tooth than that of *H*. *weihoense*. The fossette complication of hipparion largely depends on wear stage. The ontogenetic sequence analysis of Li et al.^19^ clearly shows that fossette ornamentation of *H. weihoenese* (their *Hipparion chiai*) would be simpler in very early and late stages than those in other stages. Similar analysis by Bernor and Sun^20^ found the same trends in other taxa, such as *Plesiohipparion* and *Proboscidiparion*. We have found that the Lantian specimens Liu et al. attributed to *H. chiai* are either young adult (IVPP V 3117.0) or very old (IVPP V 3116.4, IVPP V 3117.1) individuals. Liu et al.^13^ also highlighted that some large individuals of *H*. *chiai* have similar features on upper cheek teeth to those of *H*. *weihoense*. Moreover, in their description, *H*. *weihoense* and *H*. *chiai* both have rounded metaconid-metastylid complex, shallow linguaflexid and developed protostylid, i.e., these two species share identical feature combination in lower dentition. We compared a series of lower cheek tooth specimens, including reported *H*. *weihoense* and *H*. *chiai* ones from Lantian and new specimens from Linxia Basin, and have found no significant difference between these two taxa (SI Figs. S7 and S8). Therefore, *H*. *weihoense* and *H*. *chiai* are better to ascribed to the same species, *H*. *weihoense*.

Morphological comparison.

Woodburne and Bernor^21^ defined superspecific/infrageneric units for the genus *Hipparion* in the Old World. They divided the genus into four groups, mainly based on facial morphology. Group 1 consists of all the primitive forms from the Vallesian period, and some Turolian period ones. Qiu et al.^8^ argued that subgenus *Hippotherium* was equivalent to Group 1 of Woodburne and Bernor^21^. They ascribed all the Bahean forms in China and European/North African forms, such as *Hipparion primigenium*, which is common species in Europe, *Hipparion africanum* found in Bou Hanifia, Algeria, and *Hipparion catalanicum* from Hostalets, Spain, to this subgenus. According to the analyses presented in this study, in accordance with the most recent revision of Old World hipparionines by Bernor et al.^22^, there is no doubt that *Hippotherium weihoense*, *Hippotherium primigenium* and *Hippotherium catalanicum* are typical species of the genus *Hippotherium*. They share some marked characters such as shallow nasal notch at the level in front of P2, developed subtriangular POF with posterior pocket and clear anterior rim far from orbit, very long POB, complicated fossette ornamentation, elongated protocone, deep ectoflexid, and square angle on metastylid. Bernor et al.^23^ identified dental specimens from Vienna Basin Pannonian C as *Hippotherium* sp., and regarded it as the stratigraphically oldest (basal MN9, ca. 11.4–11.0 Ma) hipparion record in Europe. Based on their figures, this series of dentitions have the same characters as *H. primigenium* we listed above. So Pannonian C specimens can be treated as the first occurrence of *Hippotherium* in Europe. Arambourg^24^ erected a new species *Hipparion africanum* based on Vallesian material from Bou Hanifia, Algeria. Bernor & White^25^ performed morphological comparison and “Log10 Ratio” analysis on postcranial elements, based on which they revised *H. africanum* as “*Cormohipparion*” *africanum*, and concluded that “*C*.” *africanum* could not be referred to *Cormohipparion* s.s. but likely derived from *Cormohipparion* s.s. Bernor et al.^22^ considered “*C*.” *africanum* as an essential part of the *Cormohipparion* dispersal event. Bernor et al.^26^ erected a new species *Cormohipparion sinapensis* based on cranial material from Sinap, Turkey. Based on the description and figure of Bernor et al.^26^, this species has smaller size than *Hippotherium*, and in contrast to *Hippotherium*, it has less complicated upper molar plication, lacrimal bones extending about half way to the POF distal rim, which are indeed more similar to species of North American *Cormohipparion*^5^.

### Functional morphology

**Figure 2:**
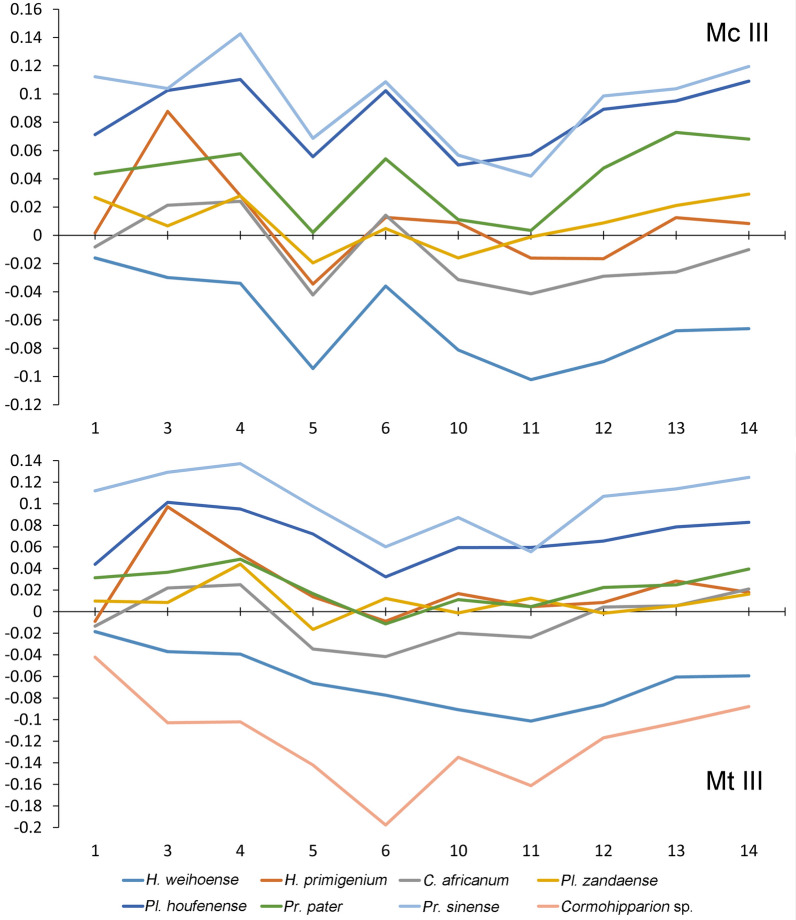
Ratio diagrams of metapodials of *H*. *weihoense* and other equids. Measurement numbers: 1, maximal length; 3, minimal breadth; 4, depth of the shaft; 5, proximal articular breadth; 6, proximal articular depth; 10, distal maximal supra–articular breadth; 11, distal maximal articular breadth; 12, distal maximal depth of the keel; 13, distal minimal depth of the lateral condyle; 14, distal maximal depth of the medial condyle. The y axis is the logarithm (base 10) of ratios between the measurements of each species and the reference species (Asiatic wild ass *Equus hemionus onager*, zero line) (generated with Microsoft Excel version 2010 by B. Sun).

**Figure 3:**
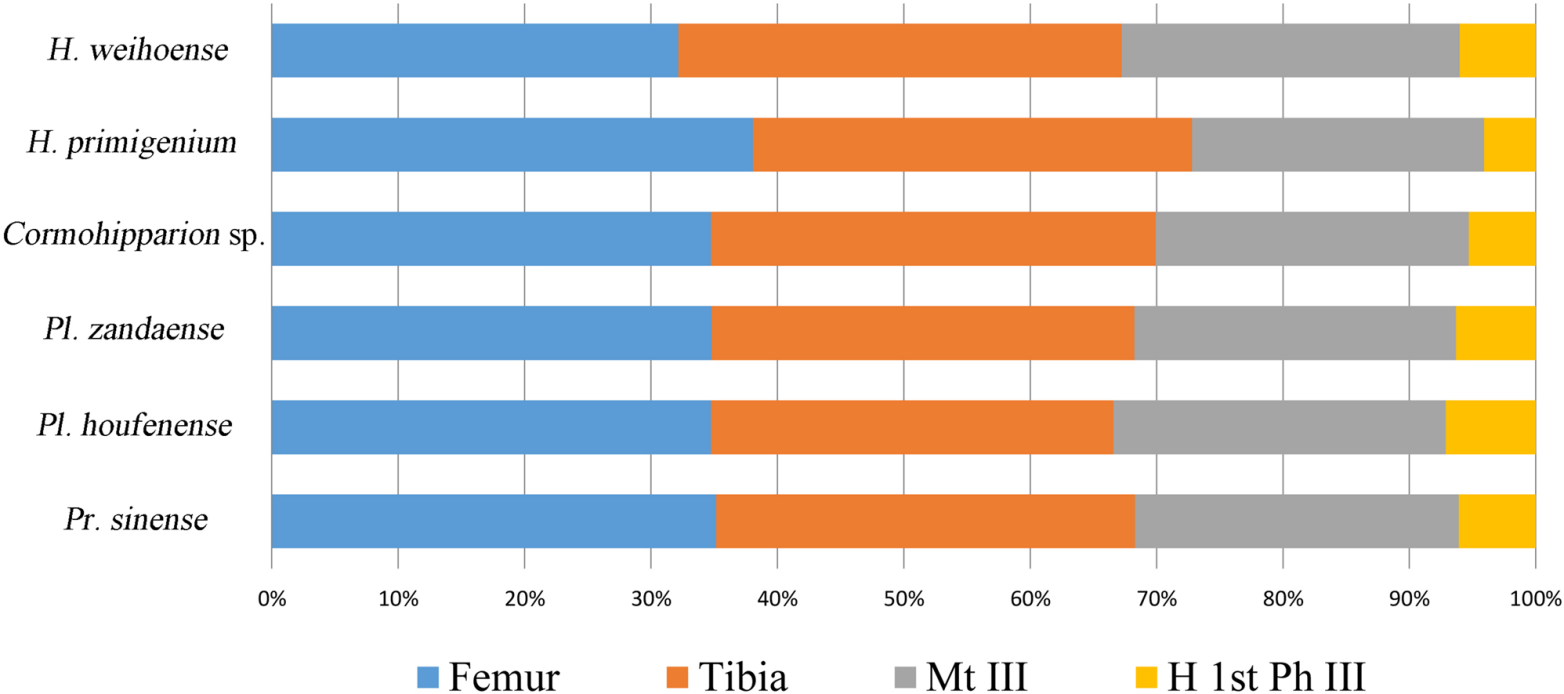
Proportions of hind limb bones in *H*. *weihoense* and other equids (generated with Microsoft Excel version 2010 by B. Sun).

The well–preserved postcranial specimens from the Niugou locality (Dashengou fauna, referred by biostratigraphic comparison of Deng et al.^17^, 9.7–8.7 Ma) indicate the locomotor ability of *H*. *weihoense*. A strong medial trochlear ridge (MTR) of the femur can fasten the medial patellar ligament, or parapatellar cartilage, and the patella when the knee joint is hyperextended^27^, forming a passive stay–apparatus to immobilise musculature in the knee extensors during long periods of standing. The femur MTR of *H*. *weihoense* is greatly enlarged relative to the lateral trochlear ridge, notably larger than in *H. primigenium*, but similar to *P. zandaense* from the Pliocene of the Zanda Basin. The ratio between the maximum depth of the MTR and the maximum length of the femur is 0.27 in *H*. *primigenium*^7^, whereas it is 0.32 in *H*. *weihoense* and 0.3 in *P*. *zandaense*^14^. Gracile limb bones are an indicator of cursorial ability, which is most clearly exhibited in the metapodials of ungulates^28^. The gracility of the metapodial midshaft is represented by diminished breadth relative to length. In Fig. 2, hipparion species are compared with extant Asiatic wild ass *Equus hemionus onager*, which is treated as standard, to show the difference in gracility of the metapodials. The ratios between the maximum length and the minimum breadth dictate that *H*. *weihoense*, *P*. *zandaense*, and *C*. *occidentale* have relatively slender metapodials (logarithm of ratio between object and standard on measurement 3 is smaller or slightly larger than that of measurement 1), but *H*. *primigenium* has very robust metapodials (logarithm of ratio between object and standard on measurement 3 is notably larger than that of measurement 1), and *Proboscidipparion* (*Proboscidipparion sinense* and *P. pater*) and *Plesiohipparion houfenense* from the North China Plain also show increased robustness. Typically, metapodial robustness of horses has been considered a marker of evolutionary grade, with slender metapodials as an advanced feature (Deng and Xue, 1999). Our results imply an exception in which metapodial robustness is considerably influenced by environmental change, represented by *P. zandaense*, which was positioned at a primitive evolutionary stage^29^ but has slender limbs, in contrast with *P. houfenense*. A high proportion of distal elements (fore and hind metapodial and phalanx) will lengthen the whole limb to keep its centre of mass situated proximally and to reduce its inertia, which allows for a long, rapid stride, as speed is the product of stride length and stride frequency^30^. Lengths of the distal elements of hindlimbs, Mt III, and the first hind phalange relative to proximal elements (humerus, radius, femur and tibia) of *H*. *weihoense* and *C*. *occidentale* are significantly longer than those of *H*. *primigenium*. Relatively elongated distal elements make the whole limb lengthened, keep the center of mass situated proximally and reduce inertia, which allows for a long, rapid stride leading to high speed^30^. So *H*. *weihoense* and *C*. *occidentale* would have stronger running ability than *H*. *primigenium*. Both the advanced *P*. *houfenense* and *P*. *sinense* have these characteristics (Fig. 3). Based on the analysis of functional morphology, *H*. *weihoense* was able to run fast and stand persistently, which is beneficial in open habitats. The running abilities of *H*. *primigenium* and *C*. *africanum* were weaker and more suited to slower movement in closed habitats^7,31^, and their locomotor function stands in contrast to the inferred ecosystem and behaviour of *H*. *weihoense*. Although no detailed measurements of *C. sinapensis* were available for analysis, Bernor et al.^26^ have stated that the limb proportions were elongate and slender compared to *Hippotherium primigenium*, which can also been see in the diagrams of Bernor et al.^26^. Therefore, *C. sinapensis* was a cursorial species, adapt to open habitat, like *H*. *weihoense.* The difference between hipparions from Asia and Europe was much influenced by environment in the early Late Miocene (see below).

## Discussion

In China, *H*. *weihoense* is mainly distributed in the Linxia Basin, Gansu^17^; the Qaidam Basin, Qinghai^32^ (9.9–7.5 Ma, biochronologic comparison by Wang et al.^33^); and Lantian^10,13^(8.21–7.26 Ma, magnetostratigraphic data of Zhang et al.^34^) and Fugu^17,18^ (7.4 Ma, magnetostratigraphic data of Xue et al.^35^), Shaanxi. The former two are located respectively on the east and north margins of the Tibetan Plateau. Deng and Wang^32^ indicated that *H*. *weihoense* (their *Hipparion* cf. *H*. *chiai* and *H*. *weihoense*) likely lived in an open habitat. Deng^36^ performed cenogram analysis on all late Cenozoic mammalian faunas in the Linxia Basin and indicated that the Bahean Guonigou and Dashengou faunas, in which *H*. *weihoense* first appeared and was dominant, both suggested open conditions. Our analysis of the locomotor ability of *H*. *weihoense* firmly supports their conclusion. The open conditions of the Qaidam and Linxia Basins imply that in the early Late Miocene the Tibetan Plateau likely had uplifted to a considerable elevation and blocked precipitation from reaching the surrounding area of the plateau, thus forming an arid, open environment. However, An et al.^37^ proposed enhanced aridity in the Asian interior at 9–8 Ma. An et al.^38^ argued that the northern part of the Tibetan Plateau had uplifted considerably in the early Late Miocene. They proposed that the northern part of the Tibetan Plateau appeared at 10–7 Ma, and that an important uplift/growth of the plateau also occurred in the same period. These events occurred significantly later than the first occurrence of hipparion horses in Eurasia. However, magnetostratigraphic investigation showed that the onset of eolian red clay deposition predated 11.4 Ma^39^. Dettman et al.^40^ indicated that a shift of carbonate δ^18^O values to − 9‰ occurred at 12 Ma, which implied a major reorganisation of atmospheric circulation patterns and a shift to more arid conditions at the NE margin of the Tibetan Plateau. Based on pollen data of Jiang and Ding^41^, the East Asian summer monsoon was generally strong between 20.1 and 14.2 Ma, decreased between 14.2 and 11.3 Ma, and has been weaker since 11.3 Ma. These results correlate with the first occurrence of hipparion horses in Eurasia (Vienna Basin Pannonian C: 11.3 Ma^5^; 11.4–11 Ma^23^; Guonigou fauna: 11.5 Ma^16^). The considerable uplift of the Tibetan Plateau likely occurred earlier than that reported in previous research^37–41^, or aridification caused by uplift of the Tibetan Plateau influenced the surrounding area earlier. The earliest appearance of *H*. *weihoense* in the Linxia Basin occurred in the Guonigou fauna^17^ (11.5 Ma, magnetostratigraphic data of Fang et al.^16^, which was the earliest record for hipparion occurrence in Eurasia). The cenogram analysis of Deng^36^ proposed that environments were more open since the Late Miocene in the Linxia Basin, represented by the Guonigou and Dashangou faunas. These open habitats were located in the area surrounding the Tibetan Plateau, and *H. weihoense* had adapted to live in them at 11.5 Ma. Dettman et al.^40^ also confirmed that the period of greatest aridity at the NE margin of the Tibetan Plateau was from 9.6 to 8.2 Ma, correlative to the age of the Dashengou fauna in the Linxia Basin, which is highly consistent with other climate records^37,38^. This was the age in which *H*. *weihoense* thrived in related areas^31,34^. Liu et al.^13^ argued that the component of the *Hipparion* fauna in Lantian, Shaanxi is an indication of a grassland environment. Deng and Wang^32^ agreed with their argument. Xue et al.^35^ argued that the Lamagou fauna (7.4 Ma) in Fugu, Shaanxi, in which *Chilotherium* and *Acerorhinus* were dominant and *H*. *weihoense* (their *Hipparion chiai*) was present, lived in an open habitat. This interpretation implies that influence of Tibetan Plateau aridification reached as far as Shaanxi. *Hippotherium weihoense* was highly adapted to open habitat and rapidly dispersed into these areas. According to paleoenvironmental data of Fortelius et al.^42,43^, the dominant climate in Turkey was arid in the early Late Miocene (11–8 Ma). The diagram of Bernor et al.^44^ showed that the metapodial of *C. sinapensis* was obviously slender compared to *H. primigenium*. Zhang et al.^45^ argued that Tibetan Plateau uplift and Paratethys retreat occurred at the same time. Retreat of the Paratethys would further reduce vapour delivery to Asia. Thus, the habitats of *H*. *weihoense* in other localities were likely similar to those of the Linxia Basin.

Based on the present study, *Cormohipparion africanum* and *H. primigenium* have relatively robust metapodials. In *H. primigenium*, the proximal elements account for a high proportion of the limbs. All of these are indicators of a closed habitat. Böhme et al.^46^ estimated precipitation for Southwest and Central Europe in the Miocene and proposed a dry period during 13–11 Ma, the time frame when the first hipparions arrived in Eurasia. More recent the environments in Europe generally became more humid, based on Fortelius et al.^42,43^, which indicates that the habitats of *H. primigenium* and *C. africanum* in Europe and North Africa, were relatively wooded during 11–8 Ma. In the same period, in the habitat of *H*. *weihoense* in northwestern China, especially the eastern margin of Tibetan Plateau, high–crowned ungulates were dominant, which indicates adaptation to an abrasive diet, which could be dominated by grasses characteristic of open environments. Böhme et al.^46^ proposed the term “washhouse climate” as an analogy for a climate under high precipitation at 10.3–9.8 Ma. This age is consistent with that of Höwenegg (10.3 Ma)^5^ and slightly younger than Eppelsheim^5^ in Germany, where specimens of *H. primigenium* were abundant.

The North American species *C*. *occidentale* also had very slender Mt III and a high proportion of the distal elements of the hind limbs (Figs. 2, 3). The habitat type at the end of the Middle Miocene and the early Late Miocene was likely open, based on the diversity and abundance of grazers in North America^47,48^. According to Mihlbachler et al.^49^, hypsodont Equinae species first occurred at 16 Ma, and became dominant at 12 Ma. Analysis of representative SEM photomicrographs of tooth microwear by Hayek et al.^50^ showed that *C*. *occidentale* were most likely grazers. Based on phylogenetic analysis, such as Woodburne^5^, *Hippotherium* was derived from the grazing *C*. *occidentale*. According to magnetostratigraphic data, the earliest species of *Hippotherium* was in Eurasia. Therefore, the origin of *Hippotherium* was likely in East Asia (not, as traditionally considered, Europe^1–5^). Based on North American palaeoecological proxies, a dominantly open habitat existed in North America significantly earlier than in Eurasia. This habitat led to the emergence of grazing *Cormohipparion* species, which would later give rise to the Eurasian hipparion horses that were adapted to open environments in the earliest phase of their dispersal. Hipparion horses in Eurasia later adapted to a variety of habitats and developed high diversity^8,22^, however retaining pre-adaptations to feeding in open environments, such as hypsodont dentitions. This is a typical example of environmental pre-adaption of late Cenozoic megaherbivores.

It is possible that two independent evolutionary routes occurred in Eurasia in the early stage of the Late Miocene: (1) the *Hippotherium* lineage including *H. weihoense* in China, *H. primigenium* in Central Europe and *H. catalanicum* in South Europe, with first occurrence at 11.5 Ma represented by *H. weihoense*; and (2) the *Cormohipparion* lineage including *C. sinapensis* in Turkey and “*C*.” *africanum* in North Africa, with first occurrence as 10.8 Ma represented by *C. sinapensis*. However, due to the scarcity of occurrences of these earliest Old World hipparionines, particularly along the possible dispersal routes via Northern Eurasia, this scenario should be seen as hypothetical and more well-dated occurrences would be needed to further test this hypothesis of the dispersal history and relationship between *Cormohipparion* and *Hippotherium* in Eurasia.

**Figure 4:**
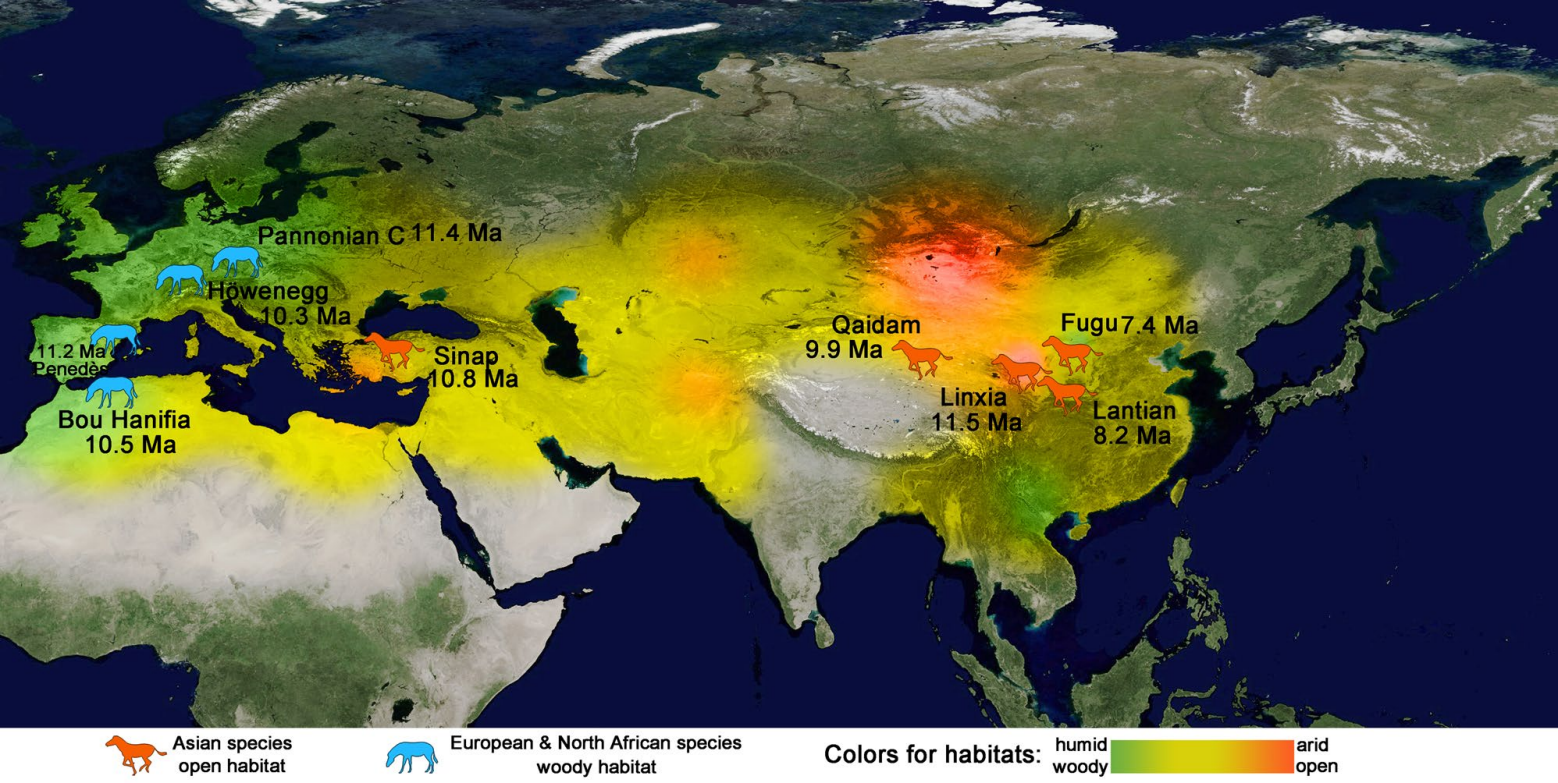
*Hippotherium* Datum and *Cormohipparon* dispersal revealed by the distribution of early Late Miocene hipparion in Old World. Environmental data after Fortelius et al.^43,44^, age data show the first appearance of hipparion in each locality, after Fang et al.^16^, Deng et al.^17^, Bernor et al.^22,23,26^, Arambourg^24^, Bernor and White^25^, Deng and Wang^32^, Wang et al.^33^, Xue et al.^35^ (generated with Adobe Photoshop version CS 5 by Y. Chen, based on original world map of GoogleEarth).

Based on the known record, *Cormohipparion* and *Hippotherium* both consisted of open-habitat forms in Asia and closed-habitat forms in Europe. The same environmental settings had a similar effect on both genera. According to the absolute age data, the occurrences of *Hippotherium* are among the earliest of all hipparionines in the Old World. This genus was widespread in Asia and Europe in early Late Miocene. The *Hippotherium* Datum, the first occurrence of *Hippotherium* in Old World, was a marked event of climatic, tectonic and biotic significance. *H. weihoense* currently seems likely to be the best candidate to represent this event in the light of available occurrences and their dating. The apparently later appearance of *Cormohipparion* in the Old World might result either from the lack of fossil evidence along the long dispersal route from North America to Western Eurasia, or it could represent a second dispersal of North American *Cormohipparion* (Fig. 4).

In summary, the following constitutes the ecological pattern of the early Late Miocene: the Tibetan Plateau uplifted and the Paratethys Ocean retreated, which aggravated aridification of mid–latitude Asia, including northwestern China and Turkey, and promoted considerable expansion of grassland. In the meantime, Europe and North Africa still had relatively closed habitats. *Hippotherium* was derived from the North American *Cormohipparion* and dispersed into Eurasia. They were highly adapted to open environments, widespread over Eurasia in the general environment of aridity. They also responded sensitively to environmental change, showed excellent adaptative ability to humid, wooded habitats in Europe and mostly more arid and open ones in Asia. The hipparionines divided rapidly into different genera with different functional morphologies to occupy diverse niches in the Old World^8,14,22^.

## Conclusion

**Figure 5:**
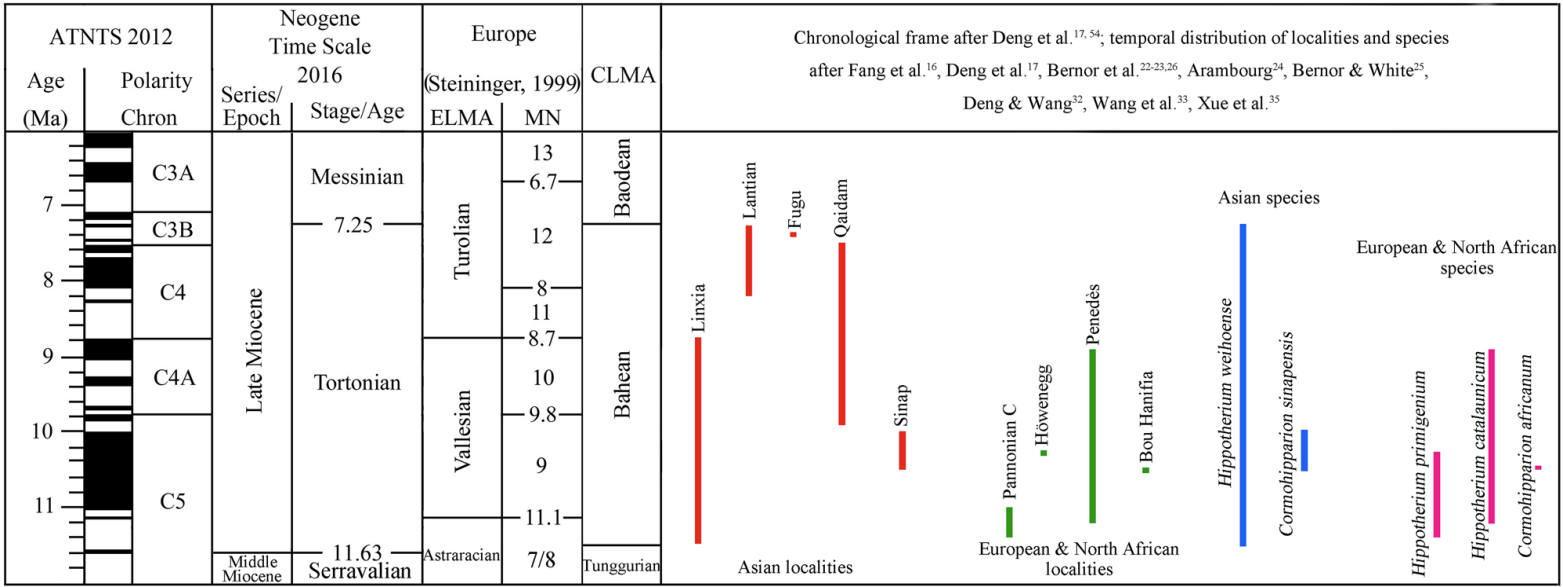
Localities and species of *Hippotherium* and *Cormohipparion* and their temporal distribution in Old World (generated with Adobe Photoshop version CS 6 by B. Sun).

- Morphologic comparison indicates previously reported *Hipparion weihoense* and *Hipparion chiai* should be referred to the same species, *Hippotherium weihoense*. This also confirms that the distribution of *H. weihoense* was throughout northwestern China.
- According to the current dating of its occurrence in East Asia, *Hippotherium weihoense* represents the earliest hipparionine in the Old World and thus marks the *Hippotherium* Datum in the early Late Miocene in Eurasia (Fig. 5).
- The first complete record of postcranial remains of *H*. *weihoense* reveals the functional morphology and the possible paleoecological behavior of this species. The locomotor comparison among *H*. *weihoense*, *H*. *primigenium* and *C*. *africanum* implies an ecological pattern in the early Late Miocene: *Hippotherium* could readily adapt to different environments and live in diverse niches.

### Terminology and measurements

The terminology of maxilla and mandible structures follows Sisson^51^ and Budras et al.^52^; detailed description on cranial and postcranial material is presented in the Supplementary Information (SI). All measurements follow Eisenmann et al.^53^, and were taken using calipers to the nearest 0.1 mm (Tables S2–S10).

### Functional morphology

We use the method of ratio diagrams of metapodials and proportions of limb bones described by Deng et al.^14^ to perform a morphological analysis on our new postcranial material (Figs. 2, 3) with the data in SI Tables S3–S6. Ratio diagrams and proportion diagrams are respectively produced with the line chart and bar chart function of Microsoft Excel.

## Supplementary Information

Supplementary Information.

## Data Availability

All data applied in the analyses in the present study are publicly available, raw date can be obtained from SI file.

## Abbreviations

HMV: Hezheng Paleozoological Museum, Hezheng, China
IVPP: Institute of Vertebrate Paleontology and Paleoanthropology, Beijing, China
AMNH: American Museum of Natural History, New York, USA
