# Supplementary material for: Hippotherium Datum implies Miocene palaeoecological pattern
Source: Sci Rep. 2022 Mar 4;12:3605. doi: 10.1038/s41598-022-07639-w (PMC8897424; doi:10.1038/s41598-022-07639-w)
Supplement: Supplementary file 1 — Supplementary Information. [file 41598_2022_7639_MOESM1_ESM.pdf]

Supplemental Information

Hippotherium Datum implies Miocene palaeoecological pattern

Boyang Sun[1,2], Yan Liu[1,2], Shanqin Chen[3], Tao Deng[1,2,4]

[1] Key Laboratory of Vertebrate Evolution and Human Origins of Chinese Academy of Sciences, Institute of Vertebrate Paleontology and Paleoanthropology, Chinese Academy of Sciences, Beijing 100044, China

[2] CAS Center for Excellence in Life and Paleoenvironment, Beijing, 100044, China

[3] Hezheng Paleozoological Museum, Hezheng, Gansu 731200, China.

[4] University of Chinese Academy of Sciences, Beijing, 100049, China

Author for correspondence:

Tao Deng

e-mail: dengtao@ivpp.ac.cn

Specimens in this study

Specimens firstly described in the present research are collected from Linxia Basin, Gansu, China (S Fig. 1-2). IVPP specimens collected from Niugou Locality including: IVPP V 24396, skull of a very old individual; IVPP V 24397, right humerus; IVPP V 24398.1－4, McIIIs; IVPP V 24399.1－2, right femurs; IVPP V 24400.1－2, right tibias; IVPP V V24401.1－9, Mt IIIs. HMV 1308, skull of a sub-adult individual; HMV 1429, skull of a juvenile individual; HMV 0476, skull of a sub–adult individule, Houshan Locality; HMV 1966, skull of an adult individual, Shuanggongbei Locality; HMV 1964, skull of an adult individual, Shuanggongbei Locality; HMV 0806, skull of an adult individual;

Description

Cranium (S Fig. 2-3). Muzzle is moderately robust. Orbits are rounded. The bottom of the nasal notch is located at the level in front of the P2. The basioccipital is narrow and high with a short and thick sagittal crest. The ventral part of the occipital condyle is moderate in length. The intercondyloid notch is narrow and has a deep groove, extending anteriorly and reaching the terminal joint of the occipital condyles. The basilar tubercles are well–developed and laterally prominent, with the midline located on the posterior side of the anterior margin of the foramen lacerum. The occipital surface is nearly pentagonal. The five sides of the pentagon are respectively two external margins of the condyles; two sides extending from the nuchal crest to the top of the paramastoid process; the posterior process of the occipital crest formed by the crossing of the sagittal crest; and the occipital crest and middle crest of the dorsal part of the nuchal ligament fossa (occipital tubercle). Occipital crest is slightly damaged; two lateral sides of the occipital are obviously constricted. Nuchal ligament fossa is deep and triangular; middle crest (occipital tubercle) and lateral ridge are strong. The foramen magnum is rounded with a width greater than the height. The supracondyloid fossa is large and deep, located laterally above the occipital condyle. The sphenoid body is narrow and high ventrally. The base of the pterygoid process is anteriorly located at the level in front of the temporal condyle. The pterygoid crest is weak and

diverges slightly laterally. The foramen lacerum is large. The upper half of the mastoid process is a strip exposed between the occipital bone and the posterior process of the squamous temporal, and the lower half is expanded to form a cylinder located at the anterolateral side of the paroccipital process, combining with the base of the paroccipital process. The central flat part of the dorsal surface of the frontal bone is narrow, and the lateral parts are inclined such that the supraorbital foramen and the upper orbital margin are lower than the frontal surface. The postorbital process is transversely wide, anteriorly concave, posteriorly convex, and relatively thin in lateral view. The free parts of the nasal bones are thin and extend anteriorly, and the posterior parts are long and strongly inclined laterally. The lacrimal sac fossa is rounded and located at the anterosuperior corner of the orbital surface. The anterior half of the upper orbital margin is a wide and deep notch. The zygomatic process of the zygomatic bone reaches the posterior orbital margin. Its ventral surface is wide, and its lateral side is a rough surface for muscular attachment. The midpoint of the posterior border of the hard palate is located at the level of the anterior margin of the M2 protocone; the anterior palatine foramen is at the level of the M2 hypocone. The pterygoid process of the pterygoid bone is low and erect, with a central groove. The preorbital fossa (POF) is strong and subtriangular with a posterior pocket and a clear anterior margin. The preorbital bar (POB) is very long. The facial crest ends above the posterior margin of P4. The infraorbital foramen is located above the P3/P4 boundary near the upper border of the maxillary bone. The facial surface of the maxillary bone is strongly constricted in front of P2 to form a large and deep buccinator fossa. The nasal process gradually becomes slender posteriorly and disappears above the midline of P2 in lateral view. I1s are both lost. I2 cup is elongated and labiolingually constricted. I3 cup is subtriangular with thin enamel.

Dentition (S Fig. 7-8)

P2. The anterostyle is short. The labial walls of the para– and metacone are flat. The plications in the fossettes are complex, especially the plis protoloph. The protocone is oval to elongated, connected with the protoloph at very late wear stage. The pli caballine is double to complex. The hypoconal groove is wide and deep; hypoconal constriction is weak to absent.

P3. The parastyle is pointed and extends labially. The labial walls of the para– and metacone are concave. The horns of the pre– and postfossette are oblique and rounded at the tip. The protocone is more elongated, rounded labially and flat lingually. The plications in fossettes are similar to P2 except that the plis protoloph are simpler. The pli caballine is usually bifid. The hypoconal groove is wide and deep; hypoconal constriction is usually present.

P4 is rather similar to P3 in morphology, but longer than P3 in size.

M1 and M2 are similar to P3 and P4 in morphology, but smaller in size. The plications in fossettes are more complex than the premolars. The para– and mesostyle are more slender. The hypoconal groove is more narrow and shallow.

M3 is usually the smallest cheek tooth. It is rectangular or triangular depending on wear stage. The mesostyle is short and narrow. The plications in fossettes are simple. The hypoconal groove is usually shallow, but sometimes may form an isolated circle at the very late wear stage.

p2: The metaconid is small and oval. The metastylid is large, leaf-shaped with a short stem, and extends more lingually. Linguaflexid opens towards the labial side. The linguaflexid is U-shaped. The entoconid is big and irregular. The protoconid is small and oval. The hypoconid is robust with flat lingual and labial walls. The pli caballinid is absent. The ectoflexid is shallow, and far from

the isthmus. The postflexid is elongated with a swollen bottom.

p3: The parastylid is small and simple and extends lingually. The metaconid and metastylid have square-shaped posterolingual angle. The linguaflexid is wide and U-shaped. The isthmus is slightly oblique anteriorly. The entoconid is smaller while the hypoconulid is very small. The labial wall of the protoconid is flat, and the hypoconid is elongated, with a flat labial wall. The anterior horn of the preflexid is long and extends labially, while the posterior one extends posteriorly. The postflexid is elongated. The pli caballinid is present usually in early wear stage. The ectoflexid reaches the opening of the isthmus with a tip that extends anteriorly.

p4: The p4 is similar to p3 but slightly smaller. The metastylid is slightly shorter and the ectoflexid is closer to the opening of the isthmus.

m1 is similar to m2, while the main differences between p3 and p4 are that the paraconid extends more lingually and the metaconid and metastylid have a trend to develop to subtriangular shaped.

Postcranial. Humerus (S Fig. 3 e–h). The greater and lesser tubercles on the lateral and medial sides, respectively, of the proximal extremity are nearly equally well developed. Both tubercles are separated by a sagittal intertubercular groove, which is wide and carries an intermediate tubercle. The body of the bone presents the major tuberosity on its medial surface about opposite the much more salient deltoid tuberosity on the lateral surface. The condyle laterally presents a slight sagittal ridge flanked by grooves. The epicondyles, lateral and medial, as well as the lateral supracondylar crest, are palpable. The shallow radial fossa is proximal to the condyle on the cranial surface of the bone.

Third metacarpal (S Fig.5.1). The facet for articulation with the magnum is fan–shaped. The dorsal margin is strongly convex; the volar margin is rather long and almost straight. The facet for articulation with the unciform is long and narrow. The anterior half is concave transversely; the posterior half is narrower and convex. The sagittal crest on the distal articulation surface is well developed.

Femur (S Fig. 4.1). The medial epicondyle is a strongly prominent ridge. The transition from the trochlea to the intercondyloid fossa is steep. The position of the distal supracondyloid fossa is low. The shaft inferior to the anterior part of the major trochanter is straight. The connecting line between the medial condyle and the trochlea is low and flat. The distal extensor fossa is a large and deep triangle. The medial and lateral trochlear ridges are divergent craniodorsally, and the former has a sharp hook on its anterodorsal aspect.

Tibia (S Fig. 4.2). The lower part of the tibia crest produces a medial slant because of the attached area of the semitendinosus tendon; the medial and lateral tubercles are widely separated from each other. The anterior depression for the cruciate ligamentous attachment is slightly larger than the posterior one, and both are shallow. The medial end of the ridge that separates the two depressions connects to the posterior end of the medial spine, and the lateral end connects to the middle part of the lateral spine.

Third metatarsal (S Fig. 5.2). The medial facet for the first and second cuneiforms and the lateral facet for the cuboid have angles of about 150° to the middle facet for the third cuneiform at the proximal anterior margin, and two high ridges separate the three facets. On the plantar surface of the shaft, the rough side surfaces become close to each other at the upper half of the shaft. The sagittal keel of the distal trochlea is distinctly prominent at the upper margin of the dorsal surface, above which is a large and deep depression.

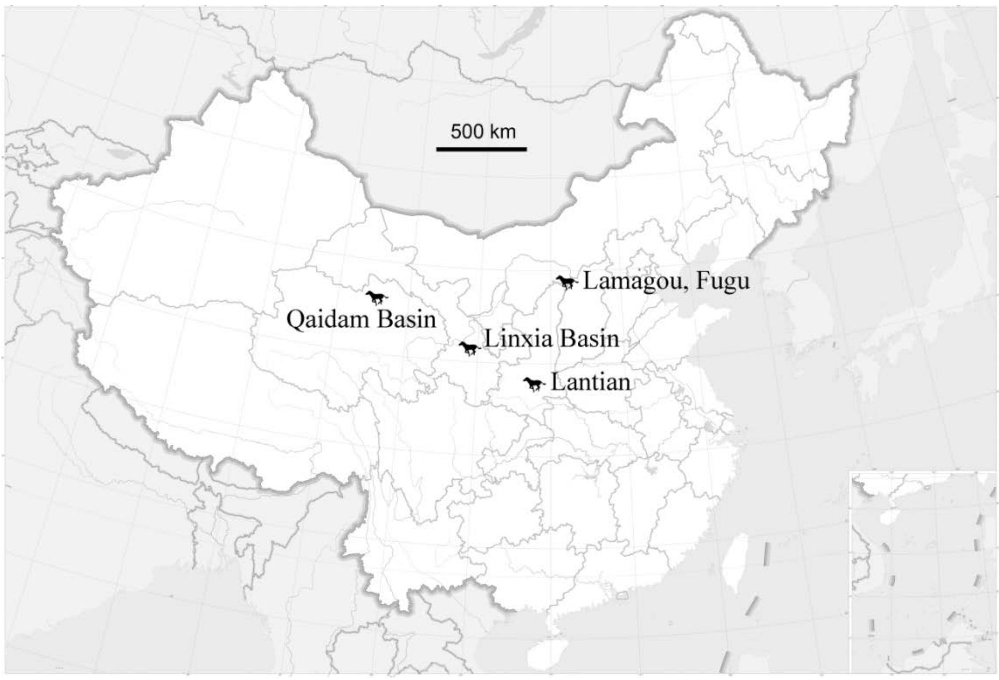

S Figure. 1. Map of locality area of *H. weihoense* in China (generated with Adobe Photoshop version CS 6 by B. Sun, based on the original map of Li & Sun[1]).

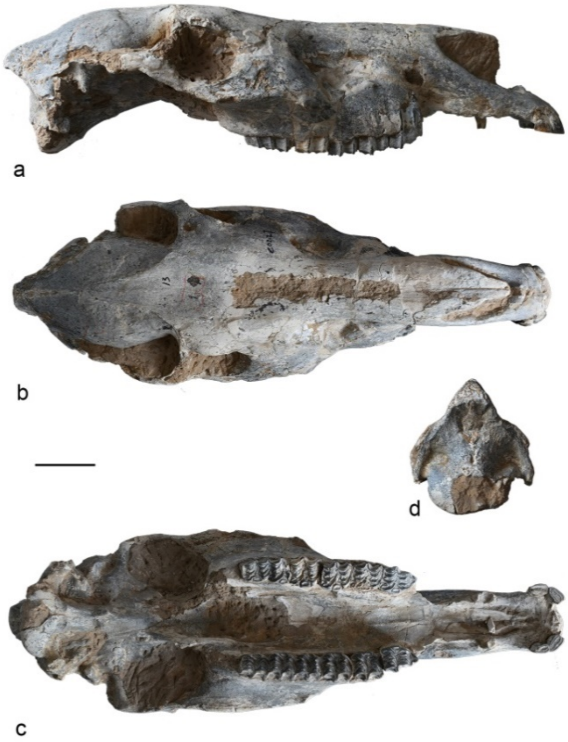

S Figure. 2. HMV 1966, skull of *H. weihoense* from Shuanggongbei locality, Linxia Basin: a, lateral view; b, dorsal view; c, ventral view; d, occipital view. Scale bar = 5 cm (generated with Adobe Photoshop version CS 6 by B. Sun, based on photographs provided by W. Gao).

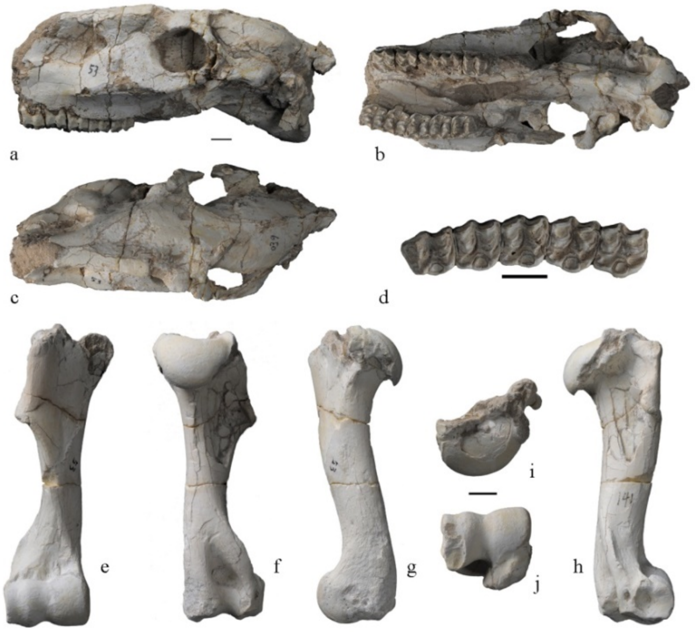

S Figure. 3. Skull (IVPP V 24396) and right humerus (IVPP V 24396) of *H*. *weihoense* from Niugou: a, lateral view; b, ventral view; c, dorsal view; d, occlusal view of cheek teeth; e, anterior view; f, posterior view; g, medial view; h, lateral view; i, proximal view; j, distal view. Scale bar = 2 cm (generated with Adobe Photoshop version CS 6 by B. Sun, based on photographs provided by W. Gao).

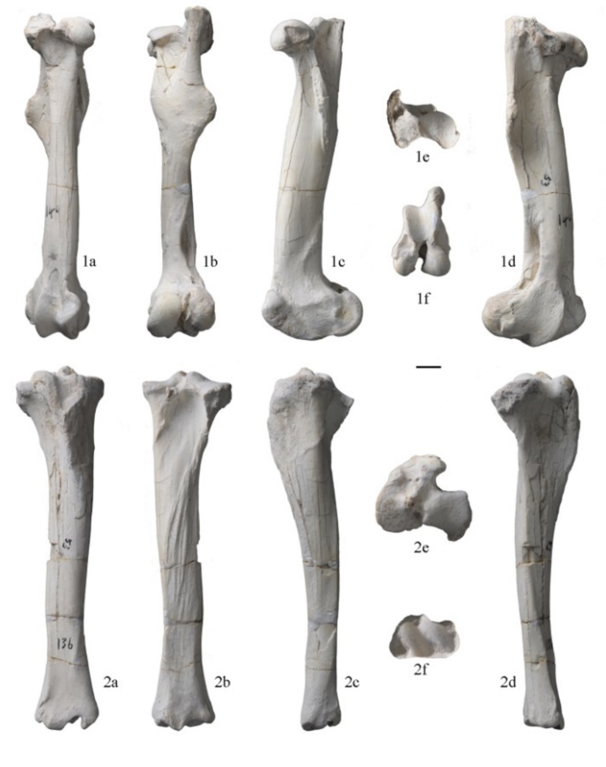

S Figure. 4. Right femur (IVPP V 24399.1, 1) and right tibia (IVPP V 24400.1, 2) of *H*. *weihoense* from Niugou: a, anterior view; b, posterior view; c, medial view; d, lateral view; e, proximal view; f, distal view. Scale bar = 2 cm (generated with Adobe Photoshop version CS 6 by B. Sun, based on photographs provided by W. Gao).

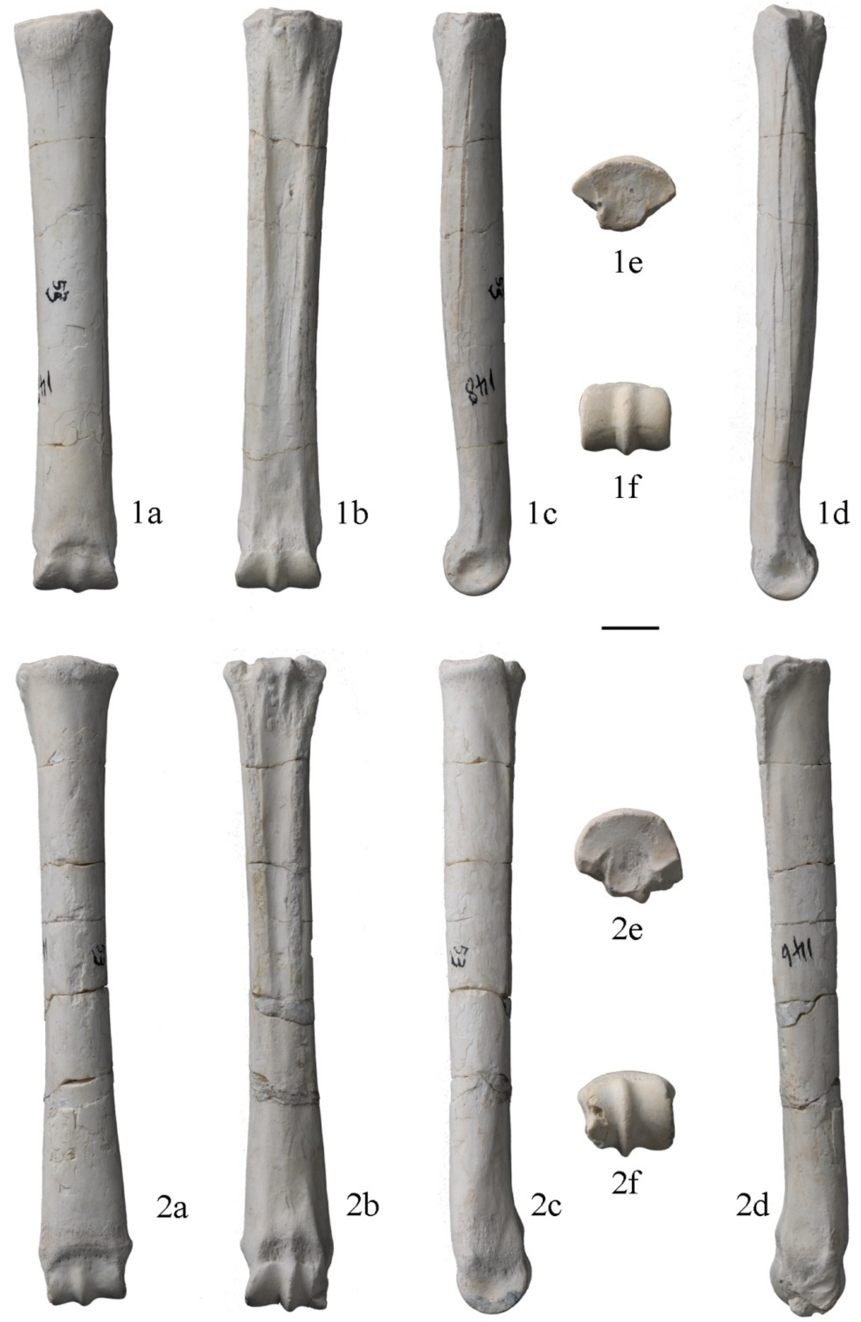

S Figure. 5. Left Mc III (IVPP V 24398.1, 1) and right Mt III (IVPP V 24401.4, 2) of *H. weihoense* from Niugou: a, anterior view; b, posterior view; c, medial view; d, lateral view; e, proximal view; f, distal view. Scale bar = 2 cm (generated with Adobe Photoshop version CS 6 by B. Sun, based on photographs provided by W. Gao).

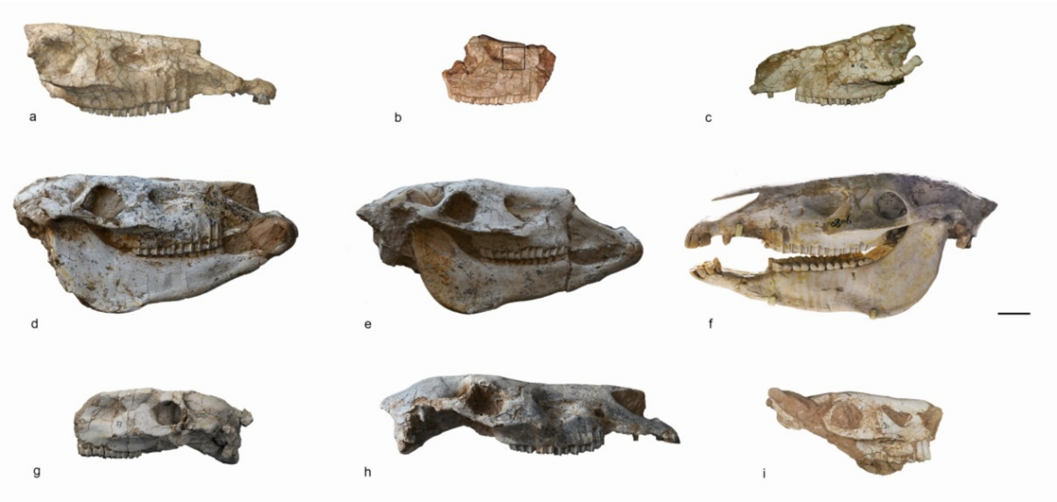

S Figure. 6. Skulls of *H. weihoense* from Lantian, Linxia Basin , a, IVPP V 3113.1, Lantian; b, IVPP V 3117.0, Lantian, previously identified as *H. chiai* by Liu et al.[2]; c, IVPP V 18793.1, Lantian; d, HMV 1963, Houshan locality, Linxia Basin; e, HMV 1967, Shuanggongbei locality, Linxia Basin; f, HMV 1968, Linxia Basin. g, IVPP V 24396, Niugou locality, Linxia Basin; h, HMV 1966, Shuanggongbei locality, Linxia Basin; i, unnumbered skull recently from Bantu localiy, Linxia Basin. Hollow square mark the part which has been deformed by diagenetically crush. Scale bar = 5 cm (generated with Adobe Photoshop version CS 6 by B. Sun, based on photographs a, d, e, f, g, h provided by W. Gao, c provided by Y. Liu, b, i provided by B. Sun).

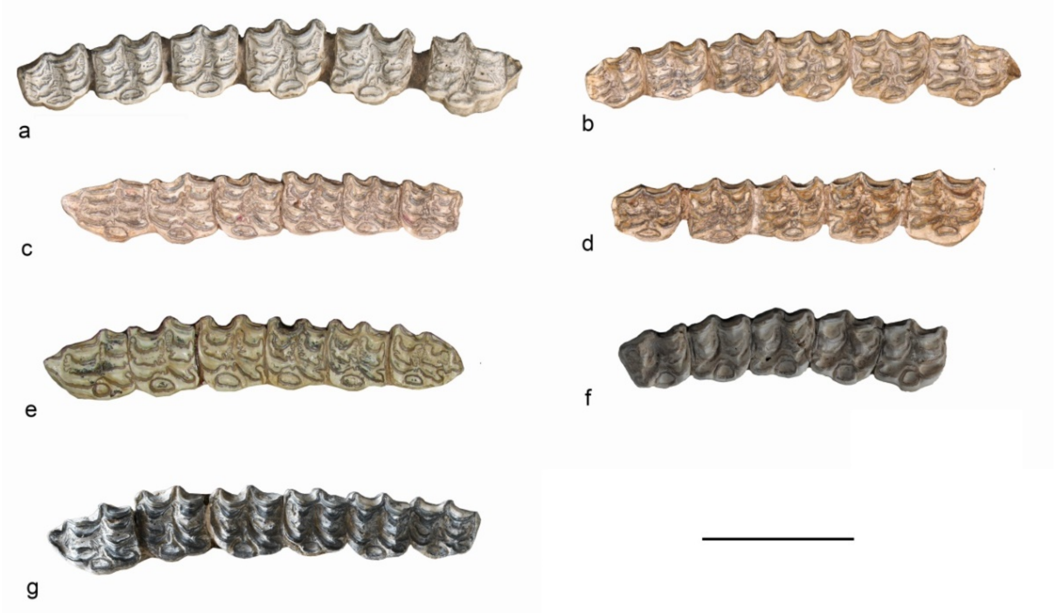

S Figure. 7. Upper cheek teeth of *H. weihoense* from Lantian and Linxia Basin and Maragheh, a, IVPP V 3113.1, Lantian, previously identified as *H. chiai* by Liu et al.[2]; b, IVPP V 3117.0, Lantian, previously identified as *H. chiai* by Liu et al.[2]; c, IVPP V 3117.1, Lantian, previously identified as *H. chiai* by Liu et al. [2]; d, IVPP V 3116.4, Lantian, previously identified as *H. chiai* by Liu et al.[2]; e, IVPP V 18793.1, Lantian; f, IVPP V 24396, Niugou locality, Linxia Basin; g, HMV 1966, Shuanggongbei locality, Linxia Basin. Scale bar = 5 cm (generated with Adobe Photoshop version CS 6 by B. Sun, based on photographs a, f, g provided by W. Gao, b, c, d, e provided by Y. Liu).

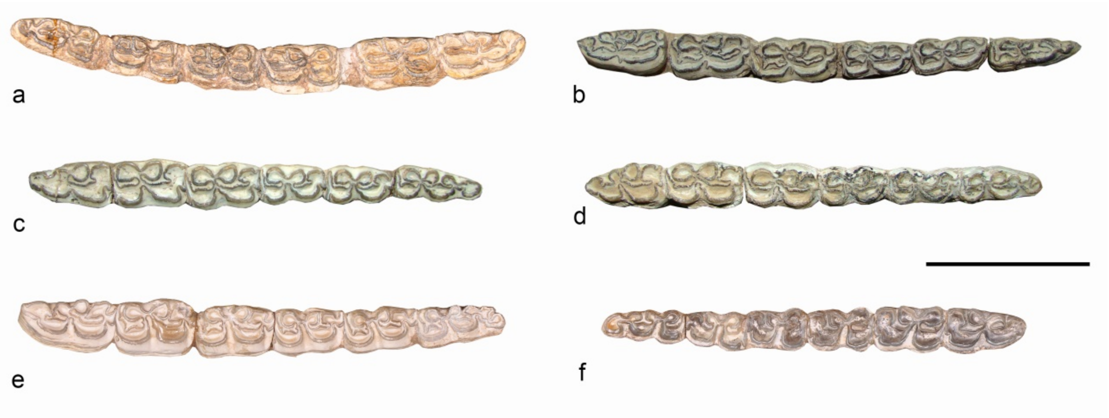

S Figure. 8. Lower cheek teeth of *H. weihoense* from Lantian and Linxia Basin, a, IVPP V 3116.4, Lantian, previously identified as *H. chiai* by Liu et al.[2]; b, IVPP V 18793.9, Lantian, modified from original picture; c, IVPP V 18793.14, Lantian; d, IVPP V 18793.11, Lantian, modified from original picture; e, IVPP V 26761, Houshan locality, Linxia Basin; f, IVPP V 26762, Houshan locality, Linxia Basin. Scale bar = 5 cm (generated with Adobe Photoshop version CS 6 by B. Sun, based on photographs a, b, c, d provided by Y. Liu, e, f provided by B. Sun).

S Table. 1. Measurements of skull of *H. weihoense* from Linxia Basin, personal measurements.

	HMV 1962	HMV 1308	HMV 1963	HMV 1967	HMV 1968	HMV 1966	IVPP V 24396
1	103	117				122	
2	90.2	111.4				106.9	
3	103.1	113.5				105	110.8
4		83.2				97.8	82.3
5		192.3				196.3	183.9
6		425		410	440	422	
7	88.9	85.8		78.4	88	79.7	
8		70.4		68.5	72.6	64	65.4
9		156.9		146	157.6	145.6	
10	58.8	63				68.9	63.5
11	22.8	27.1					20.5
12	21.6	30.4				37.8	20.5
13	34.4	54				57.4	44.2
14	31.7	30.7				35.6	
15	39.5	47.3				47	
16	70.8	75.6				72.1	65.6
17							103.2
18	115.4	141.4		130	121.8		128
19	116.8	146.9		136.1	129.8	157.4	137.7
20						58.9	
21		77.9					
22							62.9
23	284.3	333.7	321.5	335		339	
24				174.3		168.7	155.8
25	80.8	86.3	90.2			78.2	
26	93.9	98.5				92.8	102.5
28	54	59.8	59.1	56.7		57.3	57.35
29	48.4	50	47.6	49.6		44.1	47.6
30	109.8	139	127.2	141		120.3	
31	136	160.8	153.4	160.1		166.4	
32	38.9	46.8	40.8	48.4	40	41.6	45.4
33	62.4	58.8	57	62.1	63.9	60.6	62.8
34	61.5	70.9	67.8	77.6	75.6	79.6	78.2
35	40	38.6	40.7	28.6	37	40.9	40.6
36	19.1	31.6	30.6	32.8	32.1	31.3	30.6
37	29.3	47.9	51.7	45.3	46.7	40.2	41
38	53.7	75.8	80.7	82.5	84.9	74.5	79.3

S Table. 2. Measurements of upper cheek teeth of *H. weihoense* from Linxia Basin and Maragheh (mm), personal measurements.

Teeth		HMV 1962		HMV 1308		HMV 1966		IVPP V 24396
		Left	Right	Left	Right	Left	Right	Right
DP/P2	L	35.4	35.8	35.3	35.2	31	30.3	
	W	20.6	20.3	22.7	22.5	22.5	22.8	
	PL	5.4	5.5	7.3	8.4	7.3	7.5	
	PW	3.9	3.7	4.4	4.3	4.3	4.3	
	PI	15.3	15.4	20.7	23.9	23.5	24.8	
	W/L	72.2	67.3	60.3	51.2	58.9	57.3	
DP/P3	L	26.3	25.9	27.8	27.8	24	24.3	22.6
	W	19.4	19.2	24.4	24.3	23.7	23.8	23
	PL	5.1	5.1	7.3	7.9	7.3	8.1	6
	PW	4	4.1	4.1	4.2	4.4		5.5
	PI	19.4	19.7	26.3	28.4	30.4	33.3	26.5
	W/L	78.4	80.4	56.2	53.2	60.3		91.7
DP/P4	L	26.3	26.2	24.5	24.4	24.1	24.4	22
	W	17	17.5	20.4	20	25.1	25	23.8
	PL	5.2	5.6	7.4	6.6		7.1	6.8
	PW	2.8	3.3	3.5	3.5		4.5	5.8
	PI	19.8	21.4	30.2	27.0		29.1	30.9
	W/L	53.8	58.9	47.3	53.0		63.4	85.3
M1	L			23.9	24.9	21.7		20.6
	W			22.3	22.3	21.9		21.2
	PL			6.6		6.5		6.2
	PW			4		4.2		4.9
	PI			27.6		30.0		30.1
	W/L			60.6		64.6		79.0
M2	L			25.8	24.7	21.8		20.2
	W			20.3	20.4	23		21.3
	PL			7	7.3	6.7	6.8	6.1
	PW			3.9	3.5	3.9	3.9	5
	PI			27.1	29.6	30.7		30.2
	W/L			55.7	47.9	58.2	57.4	82.0
M3	L					23.9	23.8	21
	W					19.9	20.2	19.6
	PL					6.7	7.9	5.9
	PW					3.9	3.6	4.6
	PI					28.0	33.2	28.1
	W/L					58.2	45.6	78.0

S Table. 3. Measurements of proximal elements of limbs of *H. weihoense* from Niugou Locality, Linxia Basin (mm), personal measurements.

Specimen No.	IVPP V 24397	IVPP V 24399.1	IVPP V 24399.2	IVPP V 24400.1	IVPP V 24400.2
Element	right humerus	right femur	right femur	right tibia	right tibia
1			283.2	302.6	315.1
2	227.1		269	296.5	302.3
3	33.7			33.6	42.4
4	37.1			26	26.2
5	66.3		61	75.9	79.5
6		79.3	79	67.9	66
7	60		56.3	53.3	60.4
8	59.5		86.3	33.7	37.1
9	41.8		37.7	36.4	42.4
10	30.8	44	45.5		
11	36.7				

S Table. 4. Measurements and comparison of Mc III between *H. weihoense* and other equid species (mm).

	H. wei. n=5-9 (mm)	H. pri. n=10-16 (mm)	C. afr. n=2-6 (mm)	Pl. zan. V 18189 (mm)	Pl. hou. n=19-23 (mm)	Pr. pat. n=6-8 (mm)	Pr. sin. n=15-17 (mm)	E. hem. n=14-16 (mm)
1	206.9	212.8	208	225.5	249.8	234.3	274.5	212
2	202.5	207.4		217.3				206
3	23.9	31.7	27.2	26.3	32.8	29.1	32.9	25.9
4	19.5	22.5	22.3	22.5	27.2	24.1	29.3	21.1
5	34.9	39.9	39.2	41.3	49.1	43.4	50.6	43.2
6	25.2	27.9	28	27.4	34.3	30.7	34.8	27.1
7	30.1	34.6	35.7	34.7				34.2
8	8.7	11.7	10.5	11.9				12.3
9	5.2	7.5	5.85	4.5				1.9
10	32.0	39.5	36	37.3	43.4	39.7	44.1	38.7
11	30.7	37.1	35	38.4	43.9	38.8	42.4	38.5
12	24.2	28.3	27.5	30	36.1	32.8	36.9	29.4
13	20.8	24.8	22.7	25.3	30	28.5	30.6	24.1
14	22.4	26.4	25.3	27.7	33.3	30.3	34.1	25.9
15				82				
16				3.8				

Abbreviations and sources: E. hem., *Equus hemionus onager*; Pl. hou., *Plesiohipparion houfenense*; Pr. pat., *Proboscidipparion pater*; H. pri., *Hippotherium primigenium*; Pr. sin., *Proboscidipparion sinense*; Pl. zan., *Plesiohipparion zandaense*, all above after Deng et al.[3]; H. wei., *Hippotherium weihoense*; H. afr., *Hippotherium africanum*, all from personal measurements.

S Table. 5. Measurements and comparison of Mt III between *H. weihoense* and other equid species (mm).

	H. wei. n=6-10 (mm)	H. pri. n=10-16 (mm)	C. afr. n=4-8 (mm)	Cor. sp. F:AM 73903 (mm)	Pl. zan. V 18189 (mm)	Pl. hou. n=19-23 (mm)	Pr. pat. n=6-8 (mm)	Pr. sin. n=15-17 (mm)	E. hem. n=14-16 (mm)
1	235.5	242.5	240	224.6	253.2	273.8	266.1	320.3	247.5
2	231.1	237.2		219	248.4				242
3	23.5	31.4	26.4	19.8	25.6	31.7	27.3	33.8	25.1
4	23.4	28.6	26.8	20	28	31.5	28.3	34.7	25.3
5	34.8	41.8	37.4	29.2	39	47.8	42.1	50.7	40.5
6	29.9	34.3	31.8	22.2	36	37.7	34.1	40.2	35
7	26.9	39.5	35.6	27.5	37.1				36
8	9.0	9.9	8.7	7.7	7.9				8.7
9	5.7	6.5	7.2		8.6				6.2
10	31.5	39.7	36.5	28	38.1	43.8	39.2	46.7	38.2
11	29.8	37.8	35.4	25.8	38.5	42.9	37.8	42.5	37.4
12	25.3	30.7	30.4	23	30	35	31.7	38.5	30.1
13	21.0	25.3	24	18.7	24	28.4	25.1	30.8	23.7
14	23.1	27.3	27.5	21.4	27.2	31.7	28.7	34.9	26.2
15					90				

Abbreviations and sources: E. hem., *Equus hemionus onager*; Pl. hou., *Plesiohipparion houfenense*; Pr. pat., *Proboscidipparion pater*; H. pri., *Hippotherium primigenium*; Pr. sin., *Proboscidipparion sinense*; Pl. zan., *Plesiohipparion zandaense*, all above after Deng et al. [3]; H. wei., *Hippotherium weihoense*; C. afr., *Cormohipparion africanum*; Cor. sp., *Cormohipparion* sp., all from personal measurements.

S Table. 6. Measurements of Mc III of *C. africanum* in Bou Hanifia (mm), personal measurements.

	NMNH 1951-9R	NMNH 1951-9-120R	NMNH 1951-9-17R	NMNH 1951-9-26R	NMNH 1951-9-28R	NMNH 1951-9-5TypeR
1				203.6	207.6	211.8
2						203.4
3		27.2	25.8	27.0	27.2	28.6
4		22.3	21.8	22.6	22.3	22.7
5		37.2		41.0	37.2	41.3
6		26.4			27.5	30.0
7		33.4				38.0
8		11.2				9.8
9				7.2	4.5	
10	32.2	36.9	34.8	37.5	37.4	37.2
11	30.6	36.4	35.8	35.2	36.5	35.7
12	22.0	28.5	27.7	28.5	28.7	29.8
13	18.3	23.7	22.6	23.6	24.5	23.6
14	21.0	25.8	24.8	26.4	27.5	26.3

S Table. 7. Measurements of Mt III of *C. africanum* in Bou Hanifia (mm), personal measurements.

	NMNH 1951 9-914R	NMNH 1951 9-124R	NMNH 1951 9-1R	NMNH 1951 9-23TypeR	NMNH 1951 9-25R	NMNH 1951 9-72R	NMNH 90	NMNH xxxR
1	234	238	243	246	242			
2	226		241	238	237			
3	26	26	26	26	28	26		27
4	26	27	26	25	30	27		26
5	37	36	39	36	38	37		38
6	32	32	31	31	33	34		31
7	33	33	37	36	36	37		37
8	8.5			8	9.5			8.8
9	6.7	8.9			4.8	8.5		
10	35	37		38	34	37	37	
11	35	34	37	36		36	36	
12	29	30	32	30	30	31	31	
13	24	24	24	23	24	25	25	
14	26	28	28	28	28	27	28	

S Table. 8. Measurements of Mc III of *H. weihoense* in Linxia Basin (mm).

	V 24398.1	V 24398.2	V 24398.3	V 24398.4	V 3116.9	V 3141	V 18796.14	V 18792.5	V 18793.43
1	227.7	209	212.3	209.2	212.9	e198.5	206.2	e193.1	193.3
2	222.4	203.7	208.3	204.4	209.3	195.1	201.7	e189.5	187.9
3	22.1	23.3	22.6	25.3	22.2	21.4	22.1	28.8	27.7
4	19.7	18.5	19	18.6	21.3	17.5	20.4	20.1	20. 7
5	36.3	39.2	34.5	35	32.3	30.9	31.8	38.7	35.7
6	27.1	25.6	25.4	25	23.9	21.1	23.4	e29.3	25.9
7	24.6	34.7	29.6	30.7	29	27.2	27.8	35	32.3
8	8.4	9.6	9.2	9.5	7.6	7.6	7.8	9	9.7
9		6.2		5.9		4.9	5.1		4
10	31.4	32.4	32	30.2	30.5	28.9	29.5	e37.0	36.3
11	32.5	31.6	30.7	30	e29.6	27.9	28.3	34.9	28.5
12	26.4	25.2	23.6	24.1	22.4	23.6	e23.1	25.5	22.8
13	22.6	21.4	20.3	21.6	18.5	20.15	19.4	21.5	18.7
14	23.8	22.8	22.4	22.9	21.2	21.2	21.6	23.6	21.2
15									
16		6.2	7.4						

S Table. 9. Measurements of Mt III of *H. weihoense* in Linxia Basin (mm), personal measurements.

	V24401.1	V24401.2	V24401.3	V24401.4	V24401.5	V24401.6	V24401.7	V24401.8	V24401.9	V 18793.45	V 3116.9
1		249.2	236.2			226.8	238.5	239.8	237.8	225.9	e243.3
2	240.9	244.7	231.5			224.1	234.6	236.5	233.9	217.5	229.8
3	26.8	22.8	21.8	23.1	24	20.8	23.3	24	23.8	24.6	22.3
4	28.8	25.6	24.7	23.7	24.1	20	24.8	23	22.6	21.4	21.2
5		34	33.8	34.5	34.8	32.7	34.9	34.2	36.2	39.6	32.9
6	34.9		29	31.2	29	27.3	28.3	26.6	32		e30.9
7			25.9	26.6	26	25	24.8	20.5	28	36.0	29.3
8			9.2				11.3	9.1	8	8.0	e8.3
9			8.3	6.7		6.8	5.5	6	6.4	4.1	e2.0
10	36.8	32.1	29.7	31.7		28	31.1	29	34.5	e33.1	29.7
11	33	31	30.4	30.1		28	29.6	29.1	28.5	32.7	27.1
12	28.9					23.8	24.9	25.3	27.6	22.5	e23.9
13	23.4	21				20.6	21.3	21	22	17.7	20.7
14	26.3	24.7	21.2			21.6	23.4	22.2	24.9	22.4	22.4
15											

S Reference

1. Li, Y. & Sun, B. *Megantereon* (Carnivora, Felidae) in the late Early Pleistocene in China and its implications for paleobiogeography. https://doi.org/10.1016/j.quaint.2021.09.008 (2021).
2. Liu, T., Li, C. & Zhai, R. Pliocene mammalian fauna of Lantian, Shaangxi. *Prof. Pap. Stratigr. Paleont.* **7**, 149–200 (in Chinese) (1978).
3. Deng, T. et al. Locomotive implication of a Pliocene three–toed horse skeleton from Tibet and its paleo–altimetry significance. Proc. Natl. Acad. Sci. USA. 109, 7374–7378 (2012)
